# Metagenome-wide Association Studies Potentiate Precision Medicine for Rheumatoid Arthritis

**DOI:** 10.1016/j.gpb.2015.09.002

**Published:** 2015-10-15

**Authors:** Yigang Tong

**Affiliations:** State Key Laboratory of Pathogen and Biosecurity, Beijing Institute of Microbiology and Epidemiology, Beijing 100071, China

An article recently published in Nature Medicine by the group led by Drs. Jun Wang and Yingrui Li from BGI-Shenzhen and Xuan Zhang from Beijing Union Medical College Hospital, Chinese Academy of Medical Sciences revealed the relationship between the human microbiome and rheumatoid arthritis (RA) [1]. Zhang et al. performed metagenomic shotgun sequencing and a metagenome-wide association study (MGWAS) on fecal, dental, and salivary samples from a large cohort of treatment-naïve RA individuals and healthy controls. As a result, they found that the gut microbiome and the oral microbiome exhibited significant differences between RA patients and normal subjects. More importantly, the altered gut microbiome and oral microbiome of RA patients were partially corrected by disease-modifying antirheumatic drugs (DMARDs). Therefore, their findings suggest that the gut and oral microbiome composition of RA patients could be potentially used to stratify RA patients and facilitate disease diagnosis and prognosis.

RA is a relatively common autoimmune disease with high morbidity and increasing mortality, but its etiology remains obscure [2,3]. Previous studies have shown that genetic predisposition partially contributes to the disease development and some susceptibility alleles have been identified by the genome-wide association studies (GWAS) [4]. However, genetic predisposition alone cannot fully explain RA’s etiology, and environmental factors are believed to play an important role as well [3,5].

Thanks to the rapid development of next-generation sequencing (NGS) technology, especially metagenomics, the important role of microbiome has been more and more appreciated. Many diseases, especially the chronic diseases including obesity [6], diabetes [7], and autism [8], have been reported to be associated with the microbiome—the so-called “second genome of human” [9]. Microbiome is also considered to be closely related to the human immune system [10,11]. The microbial communities on human body surface (skin or mucosa) interact with the host immune system, help its maturation and enhance its defenses against harmful microorganisms [10]. On the other hand, complicated microbial antigens and enormous amount of microbial metabolites may trigger autoimmune diseases [3]. For instance, previous studies have suggested that RA could be triggered by gut microbiome [3] and gut bacterial *Prevotella copri* was believed to enhance the susceptibility to RA [12].

To understand the potential influence of microbiome on the pathogenesis of RA and explore metagenomic markers for RA diagnosis and prognosis, Zhang et al. [1] collected 212 fecal, 105 dental, and 98 saliva samples from RA patients and controls (including healthy relatives and unrelated individuals), and used shotgun sequencing technique for characterizing the microbiome in these samples. Their data revealed a clear difference in microbiome composition between RA patients and healthy controls. In addition, they also detected alterations in redox environment, transport and metabolism of iron, sulfur, and zinc in the microbiota of RA patients, indicating that the altered microbiome could play an important role in the pathogenesis of RA. Comparative analysis was also conducted on the RA patient samples collected before and after DMARD treatments. Notably, DMARD treatment not only alleviated the symptom of the RA patients, but also altered the microbiome and partially restored a healthy microbiome in the RA patients. Such alteration is most obvious in patients with the greatest clinical improvement, indicating metagenomic markers (metagenomic linkage groups, or MLGs, representing particular bacterial species) could be predictive of treatment response and thus could be used as disease classifiers. Moreover, association of the metagenomic markers from all three sites (gut, dental plaque, and saliva) provided better diagnosis of RA than just using the markers from only one site. Based on this MGWAS of oral and intestinal microbiome, classification model was constructed to diagnose and stratify RA patients.

Although the involvement of microbiomes in the pathophysiology of RA and other autoimmune diseases has been reported by many previous studies, this is the first study to compare the microbiomes from three different body sites: gut, dental plaques and saliva, of RA patients and healthy controls. Their data indicate that the abundance and alterations of the microbiome species in gut and oral samples are closely correlated with clinical manifestation and disease activity of RA. Such findings have great implications. Firstly, this study suggests that the oral and gut microbiome alterations can be of great value in RA treatment. The microbiome composition in different body sites can be potentially used as biomarkers for patient stratification as well as for diagnosis and prognosis. Secondly, this article also demonstrates the significance of MGWAS of microbiome in RA, thus providing a basis for metagenomics-assisted personalized clinical programs for RA treatment. Although the RA-associated metagenomic markers need to be validated by larger cohorts, the strategies employed in this study can be adopted to characterize the microbiome of individuals with other chronic diseases that are suspected to be related with the human microbiome, such as diabetes [7] and multiple sclerosis [13]. Lastly, this study also implies the potential of treating some chronic diseases like RA by modifying the second genome of human—microbiome, when it’s technically difficult and morally troublesome to make any changes on the human first genome—the human genome itself.

## Competing interests

The authors have declared no competing interests.
